# Airborne fungi in Longyearbyen area (Svalbard, Norway) — case study

**DOI:** 10.1007/s10661-021-09090-2

**Published:** 2021-04-23

**Authors:** Wojciech Pusz, Jacek Urbaniak

**Affiliations:** 1 Department of Plant Protection, Division of Plant Pathology and Mycology, Wroclaw University of Environmental and Life Sciences, Grunwaldzki Sq. 24a, 50-363 Wroclaw, Poland; 2 Department of Botany and Plant Ecology, Wroclaw University of Environmental and Life Sciences, Grunwaldzki Sq. 24a, 50-363 Wroclaw, Poland

**Keywords:** Airborne fungi, Svalbard, Arctic, Aeromycology, Mycology

## Abstract

Studies on the presence of atmospheric fungi in both Arctic and Antarctic polar areas are rare, and many of them were carried out briefly. Currently, when climate change is a fact, polar areas may be subject to various changes and fluctuations, negatively affecting sensitive polar ecosystems. The paper presents the results of tests on presence of fungi in the air over 30 years after the last investigations at the Svalbard Archipelago. A total of fifteen taxa of fungi were isolated in area of Longyearbyen, the majority of which were saprotrophic fungi of the genus *Cladosporium* that are associated with dead organic matter. Therefore, the presence of this taxon may be a good bioindicator of changes occurring in the Arctic environment, indirectly indicating the melting of glaciers and exposing increasingly larger areas inhabited by microorganisms, including fungi, which increase in number in the air. Additionally, the number of tourists visiting Longyearbyen is increasing, which may significantly affect the number and type of fungi in the air.

## Introduction

Fungi, a major element of atmospheric bioaerosols, are capable of existing and surviving in the air for extended periods of time (Dowd and Maier, 1999). Both the spores and the mycelium may be dangerous for people suffering from allergies, causing various health issues including asthma (Kurup et al., 2002; Asan et al., 2004). Therefore, one of the main goals of this aeromycological study monitoring fungi present in the air was to determine the quantity of fungi posing a threat to human and animal health (Asan et al., 2004; Bugajny et al., 2005; Klarič and Pepeljnjak, 2006; Topbas et al., 2006; Palmas and Cosentino, 2009; Ianovici et al., 2011; Pusz et al., 2015). Apart from its negative impact on human health, atmospheric fungi may be dangerous for plants as sources of infection (Pusz et al., 2013; Jędryczka, 2014). Moreover, fungal organisms may be capable of creating additional toxins that are harmful to humans and animals, such as endotoxins or mycotoxins (Raisi et al., 2012).

Considering this aspect, aeromycological research is considered to be very important and predicting the future symptoms of plant diseases in both crops and wild plants (Pusz et al., 2013; Jędryczka, 2014). Such research may therefore form the foundation for creating models of pathogenic spore outbreaks in plants and for studying the movement of spores and other fungal organisms across larger regions (Tomassetti et al., 2011; Leyronas and Nicot, 2012; Pusz et al., 2017). Fungi capable of travelling extensive distances with wind despite natural barriers, such as tall mountains, may be particularly relevant to understanding the role of fungi in plant disease (Nagarajan and Saharan, 2007; Vaish et al., 2011; Pusz et al., 2013; Pusz et al., 2017). Notably, the presence of numerous fungal organisms pathogenic to plants has been determined in mountainous regions.

Klarič and Pepeljnjak (2006) found spores of the genera *Cladosporium*, *Alternaria*, *Fusarium*, *Sclerotinia*, or *Botrytis* in mountainous air at 800–900 MASL in the Zagreb area. These results have been confirmed by others (Pepeljnjak and Šegvič, 2003; Magyar et al., 2012; Pusz et al., 2017). However, aeromycological studies in mountainous regions constitute a small percentage of all aeromycological research (Xia et al., 2012; Pusz et al., 2013; Pusz et al., 2017). Even fewer such studies have been conducted in polar regions where it is critical to understand the effect of climate changes taking place in these sensitive ecosystems on fungal organisms (Wookey, 2007). To date, the few studies of this kind in the Svalbard Archipelago were conducted nearly 30 years ago and were not strictly aeromycological, focusing on different aspects. Johansen and Hafsten (1988) examined the species composition of pollen in NY-Alesund and found unidentified fungal spores. The authors only identified fungal spores of the *Cladosporium* genus, which constituted less than 2% of the recorded spores.

The results were confirmed during the next air sampling cycle conducted by Johansen (1991) on Jan Mayen island. This study identified *Cladosporium* spores again, along with *Alternaria*, but over 80% of spores were unidentified. These results indicate a significant lack of knowledge about the presence of fungal spores in polar regions. While aeromycological studies have been conducted in the Antarctica, only *Cladosporium* and *Epicoccum* genera were identified in the study, which constituted a small percentage of the spores (Marshall, 1997). Aeromycological studies conducted in the Arctic Ocean by Russian researchers found thirty-nine fungal taxa occurring in the air, with the highest share being the *Cladosporium* genus. In this study, the CFU/m^3^ (colony-forming unit) was dependent on the distance from the sea; the value of colonies was smaller than 1 CFU/m^3^ on the isles and increased with the distance relative to human activity, such as in research stations (Kirtsideli et al., 2011). These results were confirmed in later studies, suggesting that under certain conditions, atmospheric fungi may cause negative health effects in humans inhabiting unfavourable areas, such as polar regions (Kirtsideli et al., 2017).

The goal of this study was to determine the species composition of airborne fungal spores in the Longyeardalen valley of the Svalbard Archipelago in May during the first thaw.

## Material and methods

### Study area

This study was carried out in the Longyearbyen area within the Svalbard archipelago, which is the northernmost (78°13 0 N, 15°33 0E) permanently inhabited city in the world. As of 2019, Longyearbyen had a population of 2.150 people. Longyearbyen has recently become a destination not only for explorers but also for leisure tourists.

According to Visit Svalbard (2021), the archipelago received 72.544 tourists in 2018 — 76% more when compared to the 41.037 tourists in 2008.

**Fig. 1:**
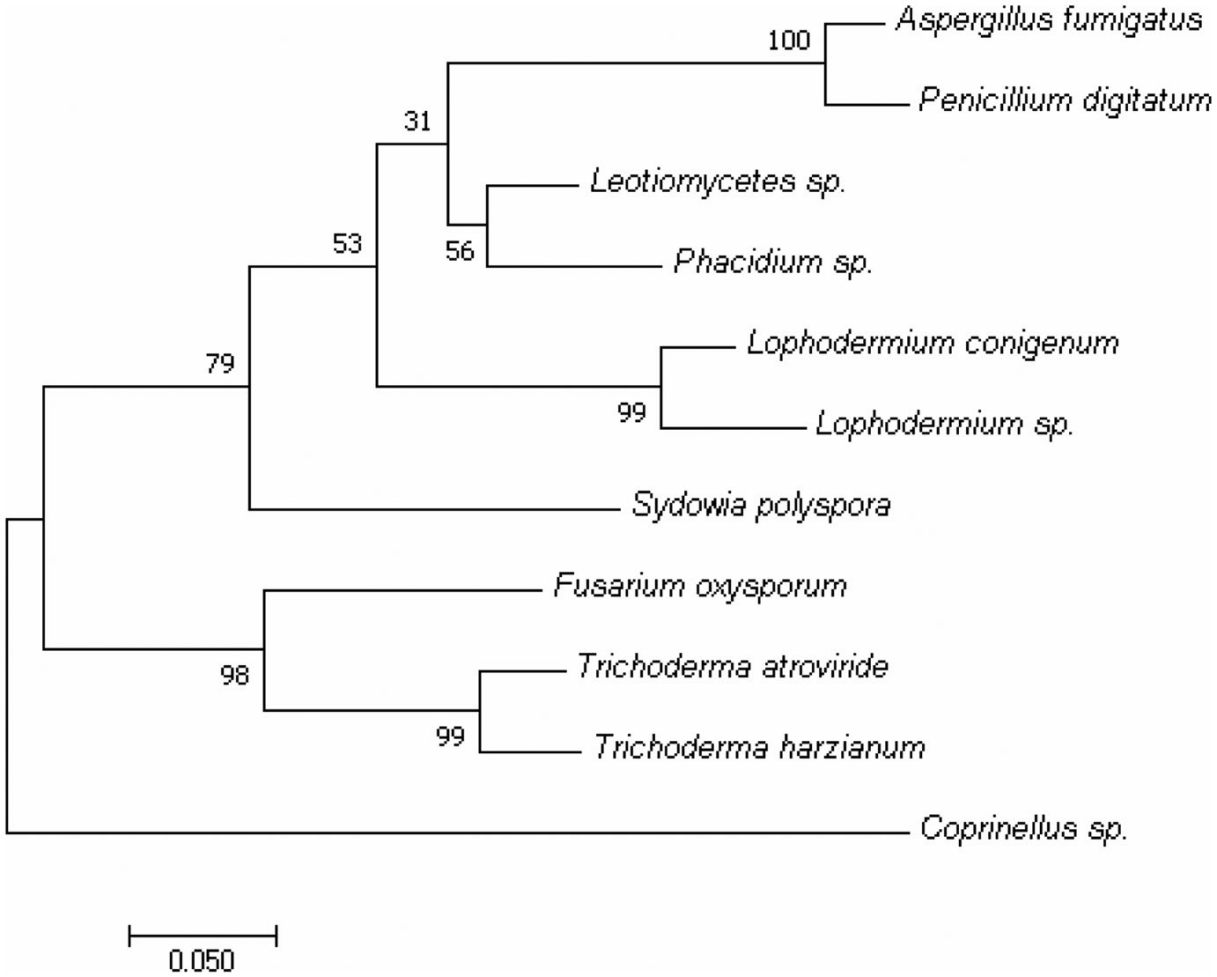
Dendrogram based on the ITS sequence of the fungi obtained from the roots of the Swiss stone pine. The evolutionary history was inferred using the Maximum likelihood algorithm in MEGA7 software. The percentage of replicate trees in which the associated taxa clustered together in the bootstrap test (1000 replicates) is shown next to the branches

Longyearbyen is located at the mouth of the Longyeardalen valley in the central part of Spitsbergen Archipelago (Szymański et al., 2019). Samples were collected at five locations in 10-km-long transect, beginning at the sea coastline, to the end of the Valley Longyearbreen Glacier. The northeast facing Longyear Glacier has an area of ~ 2.5 km^2^ and extends from ~ 250 m to over 1000 m above sea level, with a mean width of approximately 500 m (Zawierucha et al., 2019). Samples from the air were collected twice a day (8.00 a.m.–10.00 a.m. and 18.00 p.m.–20.00 p.m.) in between 14 and 24 May 2019, in two sites on Longyearbreen Glacier: in the centre of valley and at Longyearbyen centre (Fig. 1, Table 1).

**Table 1:** Sampling points

Number of point	Name of sampling point	Coordinates GPS
1	Top of Longyearbreen	78.16578 N 15.45300 E
2	Middle part of Longyearbreen	78.17437 N 15.47785 E
3	End of Longyearbyen (neighbourhood of 102 ‘Gjuestehusest’)	78.19698 N 15.55847 E
4	Middle of Longyeardalen (bridge near ‘Huset’)	78.20856 N 15.60030 E
5	Down of Longyeardalen (neighbourhood of UNIS)	78.22419 N 15.65266 E

## Sampling collection

Research was performed using an impact method and Air Ideal 3P device. By drawing an air sample, the device collects airborne fungal spores, which are then transferred into a petri dish and fed on PDA (Potato Dextrose, Agar, Biocorp) growth medium with citric acid. The device was placed 1.5 m above the ground and set to collect 100- and 150-L-volume samples. Measurements were taken in triplicate, and each sample was deposited on nine petri dishes. These were stored at room temperature (20–22 °C) over a 7 to 10 day period. The next step was to identify the species of the fungi, which was accomplished by evaluating morphological features defined by Pitt and Hocking (2009) and Watanabe (2011) and then determining the number of colonies. The number of colonies grown on each dish was calculated for 1 m^3^ of air according to the formula:

$$X=\left(a\times 1000\right)/\mathrm{V}$$

where *X* is the number of colony-forming units (CFU) in 1 m^3^ of air, *a* is the total number of fungal colonies which developed on the petri dish from airborne spores, and *V* is the volume of atmospheric air drawn, expressed in litres.

## Laboratory analysis (PCR)

In this study, molecular techniques were used for species determination. Fungal DNA was extracted with a commonly used method of nucleic acid extraction using CTAB (Doyle and Doyle, 1987). For the ITS rDNA regions, primer pairs ITS1F and ITS4 were used for species determination (White et al., 1990). The detailed PCR procedure reaction and temperature parameters were the same as those described by Pusz and Urbaniak (2017). Sequencing, post-reaction purification, and reading were performed by a nucleic acids sequencing service (Genomed S.A., Warsaw, Poland) using an ABI377XL Automated DNA Sequencer (Applied Biosystems, Carlsbad, CA, USA). Sequences were analysed with FinchTV (Patterson et al., 2004–2006) and MEGA 5.0 (Tamura et al., 2011). Species determination was verified using BLAST software (Madden, 2002), and results are presented in Table 2.

**Table 2:** Fungi species identified in air of Longyearbearn area in May 2019

Species	Isolate code	Accession number	Amplification product size (bp)	Query cover	Identities (%)	Identity with sequence from NCBI
*Cladosporium cladosporioides*	18 28	KX6777543 KX6777544	561 560	99 99	99.7 99.9	KC692219.1 KC692219.1
*Cladosporium floccosum*	4 12	KX6777545 KX6777546	560 565	98 98	99.3 99.1	MK460809.1 MK460809.1
*Cladosporium iridis*	25	KX6777547	561	95	100	KJ529007.1
*Damon diadema*	8	KX6777548	559	99	99.1	MK629946.1
*Didymella finnmarkica*	22	KX6777549	552	96	99.5	MK876388.1
*Microdochium phragmitis*	27	KX6777550	560	96	99.3	NR_132916.1
*Oidiodendron cereale*	7 7a	KX6777551 KX6777552	530 532	98 98	99.5 99.1	MH855282.1 MH855282.1
*Oidiodendron truncatum*	15	KX6777553	565	97	99.1	FJ914713.1
*Ophiocordyceps sinensis*	26	KX6777554	600	97	99.2	KT340697.1
*Phaeosphaeria culmorum*	17 14 20 21	KX6777555 KX6777556 KX6777557 KX6777558	604 606 606 602	95 97 97 98	98.9 99.2 99.2 99.7	JX981464.1 JX981464.1 JX981464.1 JX981464.1
*Phoma herbarium*	23 24	KX6777559 KX6777560	574 752	95 72	99.3 93.5	MG586981.1 MG586981.1
*Protoventuria alpina*	1	KX6777561	594	98	97.4	EU035444.1
*Sistotrema brinkmannii*	2 9 10 19 19a 16a 29	KX6777562 KX6777563 KX6777564 KX6777565 KX6777566 KX6777566 KX6777567	645 647 643 645 644 644 645	97 97 98 98 95 96 97	99.1 99.6 99.1 99.2 99.7 99.2 99.5	KF218967.1 KM232461.1 KM232461.1 KM232461.1 KM232461.1 KM232461.1 AF261656.1
*Thelebolus microsporus*	3	KX6777568	567	97	100	MF043977.1
*Vishniacozyma victoriae*	13	KX6777569	530	96	97.5	LC203739.1

## Results

During the aeromycological analyses, the highest CFU/m^3^ values were found in sampling location number four, in the ‘Huset’ region in the middle of the Longyeardalen valley. During the whole period of sample collection, nearly 4000 CFU/m^3^ was obtained, which constituted almost 90% of total CFU/m^3^ (Table 3). Behind the Longyearbyen settlement in the front of the Longyearbyen glacier, the CFU value was 370 (location number three). In the middle of the Longyearbyen glacier (location number two), the value was 80, and at the top (location number one), the value was only 10 CFU/m^3^. On the other side of the valley near the coast, the CFU value was 30 during the whole study period. The mean value of colonies found in the air in location number four was 217 per day. Higher CFU values tended to be obtained in the afternoon sample collection. During the study, 15 fungal taxa and yeast fungi were isolated from the air in the Longyeardalen valley. *Cladosporium* genus, namely *C. floccosum* and *C. cladosporioides*, characterized the highest share of the colonies: nearly 80% of the total (Table 4). *Cladosporium* spores were obtained on all surfaces studied, whereas the fungi *C. floccosum* was the only species recorded at the top of the Longyearbyen glacier, in location number one. In location number four (‘Huset’), 3050 CFU/m^3^ of *Cladosporium* genus was recorded. The same location revealed a relatively large number of the fungi *Oidiodendron cereale*, whose CFU/m^3^ value reached 190. An analysis of species isolated from the air across multiple locations showed that the highest number of species occurred in locations three and four (Table 3). Moreover, also in these locations, the number of yeast fungi has reached quantity at 230 and 470 CFU/m^3^ (Table 4).

**Table 3:** Values of CFU/m^3^ during aeromycological investigations in Longyearbyen area (CFU/m^3^) in 2019

		15.05		16.05		17.05		18.05		19.05		20.05		21.05		22.05		23.05	
	Sampling point	AM	PM	AM	PM	AM	PM	AM	PM	AM	PM	AM	PM	AM	PM	AM	PM	AM	PM
1	Top of Longyearbreen	0	10	0	0	0	0	0	0	0	0	0	0	0	0	0	0	0	0
2	Middle part of Longyearbreen	0	20	0	0	20	0	0	0	0	0	0	0	40	0	0	0	0	0
3	End of Longyearbyen	20	10	10	10	10	0	10	10	10	10	10	10	100	100	20	10	10	10
4	Middle of Longyeardalen	20	420	120	130	250	310	210	230	120	240	200	300	400	420	100	130	120	200
5	Down of Longyeardalen	0	0	0	0	0	0	0	0	0	0	0	10	10	10	0	0	0	0

**Table 4:** Occurrence of fungi on air of Longyearbyen area (CFU/m^3^)

Fungal taxa	Number of sampling points				
	1	2	3	4	5
*Cladosporium cladosporioides*		10		240	
*Cladosporium floccosum*	10	20	50	2810	10
*Cladosporium iridis*				10	
*Damon diadema*			10	20	
*Didymella finnmarkica*				10	
*Microdochium phragmitis*				40	
*Oidiodendron cereale*				190	10
*Oidiodendron truncatum*			10		
*Ophiocordyceps sinensis*				20	
*Phaeosphaeria culmorum*		10	10	40	
*Phoma herbarum*				20	
*Protoventuria alpina*			10		
*Sistotrema brinkmanni*		40	10	10	
*Thelebolus microsporus*			20	40	10
*Vishniacozyma victoriae*			20		
Yeasts colonies			230	470	
Total value of CFU/m^3^	10	80	370	3920	30

## Discussion

[Historically, detailed aeromycological studies have a long tradition of being conducted in polar regions, though for many years, they were conducted rather superficially (Johansen and Hafsten, 1988; Johansen, 1991). Research of this kind is underappreciated and underutilized in rapidly changing polar environments (Wookey, 2007; Wojtuń and Roniker, 2018). Moreover, microorganism diversity, including diversity of fungi, may directly indicate the abundance of species in polar regions; therefore, ecological studies of microorganism groups should be conducted, including various taxonomic groups (Wutkowska et al., 2018). Aeromycological research should be a focus of future studies, since this approach can answer questions about the spreading of spores and the concentration of colony-forming fungi in regions of interest, including in extreme climates (Pusz et al., 2017).

Our findings highlight this fact, even though they significantly differ from the results of aeromycological studies conducted 30 years ago. The majority of mycoaerosol collected in the Longyearbyen area constituted of fungi of the *Cladosporium* genus (nearly 90%), whereas Johansen and Hafsten (1988) recorded only 2% of *Cladosporium* spores in the total mass of spores obtained in the research station in NY-Alesund. A slightly higher percentage of *Cladosporium* genus fungi was recorded by Johansen (1991) on Jan Mayen isle, where these spores constituted nearly 5%. In that particular case, the sample collection period may be significant due to the start of melting snow in arctic summer and uncovered soil surface.

Since *Cladosporium* fungi are saprobiontic and are weak plant pathogens (Ogórek et al., 2012) connected with organic matter and is more prevalent in polar summer in the Arctic than in thawing periods during rising temperature. These conditions occurred this year in May during the experiment. Therefore, it is possible that the higher proportion of *Cladosporium* fungi in the Svalbard Archipelago air may indicate changes in that environment. Glacier thawing and their regression, shorter ice cover presence, and temperature increase are factors influencing the colonization of post-glacial areas by microorganisms, including fungi (Grzesiak i Zdanowiski, 2013). This process is reflected in the current study, where the highest species abundance and the highest concentration of fungi spores were recorded in the middle of the Longyeardalen valley (location number four). During the experiment in May, snow at the location thawed rapidly revealing Arctic tundra. This is correlated with soil science research conducted earlier in Longyearbyen. The soil in the middle of the valley is characterized by a larger share of loamy and clayey fractions, as well as higher organic matter, organic carbon, and nitrogen contents (Szymański et al., 2019). Consequently, the soil is darker and heats faster, which, combined with faster thawing and high organic matter content, results in an increase in microorganism richness, including fungi. This is further indicated by the species composition of fungi, most of which are saprobiontic (Rosa et al., 2019).

Accordingly, aeromycological research could be an appropriate addition to complex ecological studies of polar regions. Fungi constitute a significant element of this environment, and their role in polar regions, including energy circulation, is not fully know yet, despite being more significant than we tend to think (Dresch et al., 2019). This study allows us to monitor the influence of fungi on the Arctic ecosystem and its inhabitants, particularly people who are living in the Arctic (Kirtsideli et al., 2017).

## Conclusion

Our results indicate that the main source of airborne fungi biomass is organic material accumulated under snow. The tundra, which appears when the snow is melting, is a feeding place for animals including birds and reindeers, which also indirectly increases the concentration of fungi in the air, as well as the species richness of mycobiota. Snow yields are correlated with soil type due to organic matter content and soil colour. Compared to previous aeromycobiotic research studies conducted at Svalbard nearly 30 years ago, it is clear that there are changes taking place in the polar environment. Research studies conducted during snow melting may be compared to similar research carried out in the middle of the polar summer, taking into account the increased tourism during this period. Furthermore, the high number of tourists visiting Longyearbyen may affect the species composition and concentration of fungal spores present in the air.

## Data Availability

The datasets generated during and/or analysed during the current study are available from the corresponding author on reasonable request.
